# Method to quantify live and dead cells in multi-species oral biofilm by real-time PCR with propidium monoazide

**DOI:** 10.1186/2191-0855-3-1

**Published:** 2013-01-04

**Authors:** Gerard Àlvarez, Marta González, Sergio Isabal, Vanessa Blanc, Rubén León

**Affiliations:** 1 Department of Microbiology, DENTAID S. L., Ronda Can Fatjó 10, Parc Tecnològic del Valles, Cerdanyola, 08290, Barcelona, Spain

**Keywords:** Oral biofilm, Real-time PCR, Propidium monoazide (PMA), Antiseptic

## Abstract

Real-time PCR (qPCR) is a widely used technique in analysing environmental and clinical microbiological samples. However, its main limitation was its inability to discriminate between live and dead cells.

Recently, propidium monoazide (PMA) together with qPCR has been used to overcome this problem, with good results for different bacterial species in different types of samples.

Our objective was to implement this technique for analysing mortality in multi-species oral biofilms formed *in vitro* with five oral bacteria: *Streptococcus oralis*, *Streptococcus gordonii*, *Veillonella parvula*, *Fusobacterium nucleatum* and *Prevotella intermedia*. We also tested its effectiveness on biofilms treated with an antiseptic solution containing 0.07% w/w cetylpyridinium chloride (CPC).

Standardisation of the qPCR-PMA method was performed on pure, heat-killed planktonic cultures of each species, detecting mortality higher than 4 log in *S. oralis*, *S. gordonii* and *F. nucleatum* and higher than 2 for *V. parvula* and *P. intermedia*. We obtained similar results for all species when using CPC.

When we analysed biofilms with qPCR-PMA, we found that the mortality in the non-CPC treated multi-species biofilms was lower than 1 log for all species. After treatment with CPC, the viability reduction was higher than 4 log in *S. oralis* and *S. gordonii*, higher than 3 log in *F. nucleatum* and *P. intermedia* and approximately 2 in *V. parvula*.

In short, we standardised the conditions for using qPCR-PMA in 5 oral bacterial species and proved its usefulness for quantification of live and dead cells in multi-species oral biofilms formed *in vitro*, after use of an antiseptic.

## Introduction

Culture and real-time PCR (qPCR) are the methods most used for quantitation of microorganisms in different conditions or environments (Sontakke et al. 2009; Maciel et al. 2011). However, these techniques have limitations in that they tend to underestimate or overestimate microbial counts. Culture methods only allow for counting viable cells that are capable of forming colonies on nutrient media, without detecting dead cells, viable but non-culturable bacterial cells (VBNC) and those that require special growth conditions (Oliver 2005; Cerca et al. 2011). On the other hand, qPCR detects all cells in a sample, including the dead cells or the DNA of some of them that can be found in the environment (Pathak et al. 2012).

In recent years, a new intercalating agent has been used together with qPCR reaction to discriminate and count both live and dead cells in a microbiological sample. This new methodology is based on the use of propidium monoazide (PMA), a derivative of propidium iodide (PI) commonly used in microscopy and flow cytometry to stain dead cells. Once it penetrates the membranes that have lost their integrity, PI binds to the dsDNA, and if the dsDNA-PI complex is excited with a wavelength of 493 nm, it emits a fluorescence of 630 nm (colour red). The PMA maintains the same characteristics as PI, and if the dsDNA-PMA complex is photoactivated (using bright visible light), the monoazide group is converted to a highly reactive nitrene radical which readily reacts with any hydrocarbon moiety to form a stable covalent bond (Nocker et al. 2006).

This permanent modification of the DNA, theoretically prevents its use as a template for PCR reaction. Therefore, pre-treatment of a sample with PMA prevents amplification of dead cells DNA, allowing the qPCR method to quantitatively discriminate between live and dead cells.

The oral cavity has a complex microbiota consisting of more than 700 bacterial species. While oral bacteria can be found in planktonic form in saliva, their main form of growth in the different oral niches is in a biofilm structure (Kolenbrander et al. 2002). This form of life provides them with a sheltered environment, for example, from disinfecting treatments with antibiotics or antiseptics (Costerton et al. 1999; Guggenheim et al. 2001), which explains why mechanical therapy is the most efficient treatment for periodontitis (Herrera et al. 2008).

The most common study techniques of oral microbiota in conditions of health or disease have included culture, PCR (and qPCR) and checkerboard methods. The limitations of these methodologies were previously mentioned, and DNA-DNA hybridization has the same limitations as qPCR and if its conditions are adjusted, at most it can be used as a semi-quantitative technique.

The use of real-time PCR and PMA has been effectively evaluated for different microorganisms of environmental and clinical interest. This method has emerged as an effective tool to quantify and discriminate viable and non viable cells in fastidious or very slow growing organisms (Kralik et al. 2010). It has also been used in samples containing fungi (Vesper et al. 2008), spores (Rawsthorne et al. 2009), viruses (Fittipaldi et al. 2010) and protozoa (Fittipaldi et al. 2011).

Recently, Loozen et al. 2011 used this methodology to quantify live and dead cells in planktonic oral bacterial samples, however, the differentiation between viable and non-viable bacteria when they form part of a multispecies oral biofilm has not been tested with this methodology. The purpose of this study is, therefore, to evaluate whether PMA pre-treatment can be used with qPCR to quantify bacterial viability in a multispecies oral biofilm formed *in vitro*.

## Materials and methods

### Bacterial strains and growth conditions

The bacterial strains used in this study comprise: *Streptococcus oralis* CECT 907 T, *Streptococcus gordonii* ATCC 49818, *Veillonella parvula* ATCC 10790, *Fusobacterium nucleatum* DSM 20482 and *Prevotella intermedia* NCTC 13070.

All species were grown on non-selective blood agar plates (No. 2 of Oxoid; Oxoid Ltd, Basingstoke, UK), with 5% defibrinated horse blood, hemin (5 mg/l) and menadione (1 mg/l) and incubated at 37°C under anaerobic conditions. Liquid cultures were prepared from single colonies transferred to modified brain heart infusion (brain heart infusion broth [37 g/l], mucin from porcine stomach type III [Sigma-Aldrich Chemie GmbH, Buchs, Switzerland] [2.5 g/l], yeast extract [1 g/l], L-cysteine [0.1 g/l], sodium bicarbonate [2 g/l] and supplemented with hemin [5 mg/l], menadione [1 mg/l] and glutamic acid [0.25%]) and grown anaerobically at 37°C.

### Biofilm formation

Biofilms were prepared as previously described by Sánchez et al. 2011. Briefly, they formed biofilms on ceramic calcium hydroxyapatite discs (HA), 7 mm in diameter and 1.8 mm thick (Clarkson Chromatography Products, Williamsport, PA, USA) within the wells of presterilized polystyrene 24-well cell culture plates (Greiner Bio-one, Frickenhausen, Germany). In these wells 1.5 ml bacterial pools were placed after being previously adjusted in order to obtain a solution containing 10^3^ CFU/ml for *S. oralis* and *S. gordonii* and 10^6^ CFU/ml for *V. parvula, F. nucleatum* and *P. intermedia*. This was then incubated in anaerobic conditions for 4 days, with a change to fresh medium at 48 h intervals.

The thickness and structures of biofilms were analysed through a Live/Dead BacLight bacterial viability stain kit (Molecular Probes BV, Leiden, the Netherlands), and were observed under a LEICA SP5 confocal microscope (Leica Microsystems, Heidelberg, Germany). During the microscopy analysis, the biofilms were kept in 5% CO_2_ at 37°C.

### Planktonic cultures for killing conditions

Each species was prepared as described above and grown until they reached the log phase. The bacterial concentration was adjusted by measuring optical density at 550 nm to obtain bacterial suspensions with concentrations of 10^8^ CFU/ml for the five species. In each case the CFU/ml was determined in triplicate by plating 100 μl aliquot from serial dilutions on non-selective blood agar plates.

The cells were pelleted and washed once with PBS (NaCl 137 mM, KCl 2.7 mM, Na_2_HPO_4_ 10 mM, KH_2_PO_4_ 2 mM, pH 7.4) and aliquoted in 1.5 ml microcentrifuge-tubes.

As a positive control, cells were initially killed by exposure to heat or isopropanol, and the same results were obtained (data not presented). From these results, heat-killing conditions were preferred due to its ease of use. In this last case, five hundred microliters of cell suspension were killed by exposure to 90°C for 15 min using a standard laboratory heat block. Furthermore, as a killing method cells were also tested against a CPC solution (0.07% w/w in water) for 1 min.

In all cases the absence of viability was verified using plate culture, followed by incubation at optimal conditions.

### Biofilm killing conditions

Before treatment, mono and multi-species biofilms were gently washed with PBS to detach cells not forming part of the biofilm and to remove the culture medium. The discs were immersed in the CPC solution for 5 min, washed in 1 ml of PBS (to eliminate the CPC) and subsequently vortexed for 5 min in 1 ml of PBS. Lastly, 100 μl were serially diluted and plated on blood agar. Four replicates were performed.

### Optimisation of PMA treatment

The procedure was adapted from previous studies of Nocker et al. 2006 and Nocker et al. 2007. Briefly, PMA (Biotium, Inc., Hayward, CA, USA) was dissolved in 20% of dimethylsulfoxide (DMSO) (Sigma). Both heated and unheated suspension cells were reacted with PMA at different concentrations, 50, 100 and 200 μM. PMA was added to 500 μl of heat-killed and live cells and kept in light-transparent 1.5 ml microcentrifuge-tubes. As controls, identical volumes of 20% de DMSO (without PMA) were added to 500 μl of heat-killed and live cells. Sample tubes were immediately incubated with PMA in the dark with occasional thorough mixing, being also tested at different time periods: 5, 10, 15 and 30 min. Later the samples were light-exposed for 5, 10 and 30 min, using a 650-W halogen light source (230 V, GX6.35 FS1, 3400 K; Osram GmbH, Augsburg, Germany) at a distance of 20 cm. The sample tubes were placed horizontally on ice and were occasionally mixed to guarantee homogenous light exposure. Finally, the best conditions encountered for the five species were: 10 min of incubation in the dark with PMA 100 μM and 5 min of light exposure (data not presented).

### DNA extraction methods and qPCR conditions

The DNA isolation was performed using the QiAamp DNA Mini Kit (Qiagen) following manufacturer’s instructions, with some modifications. Briefly, after photo-induced cross-linking, cells were pelleted at 12,000 × g for 4 min, suspended in 180 μl of a 20 mg/ml lysozyme solution (20 mM Tris·HCl, pH 8.0; 2 mM EDTA; 1.2% Triton X-100) and incubated 30 min at 37°C. Thereafter, 200 μl of Buffer AL (provided in the kit) and 20 μl RNase A (20 mg/ml) were added and incubated 20 min at 56°C, followed by 10 min at 70°C with 20 μl proteinase K (20 mg/ml). Further procedure was conducted according to the manufacturer’s protocol. Lastly, DNA was resuspended in 100 μl of buffer AE (provided in the kit), visualised in 0.5% agarose gel and quantified using Nanodrop® ND-1000 UV–vis spectrophotometer (Nanodrop Technologies, Wilmington, DE USA).

Quantitative PCR was performed using a LightCycler® 480 II (Roche Diagnostics, Penzberg, Germany). Specific primers (Invitrogen Life Technologies, Carlsbad, CA, USA) and Taqman probes (Applied Biosystems, UK and Roche Diagnostics, S. L) selected for each species are showed in Table 1. In order to establish the amplification conditions, different concentrations of primers and probe were tested, performing the reactions in a 20-μl volume, containing LightCycler® 480 II Probes Master (Roche Diagnostics), 0.8 μM of each primer, 0.2 μM of Taqman probe, 5 μl of isolated genomic DNA and PCR grade sterile water. Each set of samples included a PCR grade sterile water control. Data analysis and crossing point (Cp) were calculated by LightCycler® 480 Software 1.5 (Roche Diagnostics) using the second derivative maxim method. The qPCR reaction was conducted using an initial cycle of 95°C for 10 min, followed by 40 cycles of denaturation at 95°C for 10 s, annealing for 30 s, and extension at 72°C for 1 s. The temperature of annealing depended on the species, and was 65°C for *S. oralis*, 60°C for *V. parvula*, *F. nucleatum* and *S. gordonii*, and 58°C for *P. intermedia*. In each reaction, positive and negative controls and test samples were performed in triplicate.

**Table 1:** List of primers and TaqMan probes for each targeted oral species with expected amplicon sizes

**Primers or probes**^**a, b**^	** Sequence (5**^**′**^**➞3**^**′**^**)**	**Product size (bp)**	**Source**
* S. oralis*			
gtfR F	ACCAGCAGATACGAAAGAAGCAT	235	This study
gtfR R	AGGTTCGGGCAAGCGATCTTTCT		
TaqMan probe	AAGGCTGCTGTTGCTGAAGAAGT		
*S. gordonii*			
gtfG F	CGGATGATGCTAATCAAGTGACC	177	This study
gtfG R	GTTAGCTGTTGGATTGGTTGCC		
TaqMan probe	AGAACAGTCCGCTGTTCAGAGCAA		
*V. parvula*			
16S rRNA F	GGATAGATGAAAGGTGGCCTCT	72	This study
16S rRNA R	CCAACTAGCTAATCAGACGCAAT		
LNA probe*	FAM-GAAGGAGG		
*F. nucleatum*			
fadA F	TGCAGCAAGTTTAGTAGGTG	146	This study
fadA R	CATTGTAAACTTGTTCATTTTGT		
TaqMan probe	AGCACTAGATGCTGAATACCAA		
*P. intermedia*			
16S rRNA F	CGGTCTGTTAAGCGTGTTGTG	100	Loozen et al.
16S rRNA R	CACCATGAATTCCGCATACG		
TaqMan probe	TGGCGGACTTGAGTGCACGC		

a) F, Forward primer; R, Reverse primer.

b) All TaqMan probes correspond to: 5^′^ FAM, 3^′^ TAMRA.

* LNA probe, Roche Diagnostics.

Standard curves were developed for the qPCR of each bacterial species using as a template known concentrations of genomic DNA. Serial 10-fold dilutions in PCR grade sterile water were made to prepare standard DNA ranging from 10^2^ to 10^9^ cells. Standard curves were constructed by plotting Cp values *vs* log cells and used to quantify the number of cells of each species, as required, based on their respective Cps. When analysing the samples, each run of qPCR was conducted with standard DNA curve.

The following was therefore estimated:

(1) Logarithm of the total number of cells: qPCR without PMA

(2) Logarithm of the number of live cells: qPCR-PMA

(3) Logarithm of the number of dead cells: (Δlog_10_ cells): qPCR – (qPCR-PMA)

### Statistical analysis

Means, standard deviations and *p*-values were calculated by using the Microsoft Excel software (Microsoft, Redmond, WA, USA). Equations of standard curves, coefficients of determination (R^2^) and efficiencies (E) (Table 2) were obtained by means of LightCycler® 480 Software 1.5.

**Table 2:** Linearity of qPCR calibration curves

**Species**	**R**^**2**^	***E***
*S. oralis*	0.999	0.915
*S. gordonii*	0.998	0.920
*V. parvula*	0.999	0.950
*F. nucleatum*	0.999	0.950
*P. intermedia*	0.999	0.950

R^2^ indicates the regression coefficient. PCR efficiency (*E*) is obtained from *E*= 10^-1/*s*^ – 1, where *s* is the slope of the regression line between Cp values and log cells (Knutsson et al., 2002).

Cp values *versus* log (number of cells) of five oral bacterial species.

Means of non-PMA treated and PMA treated samples were compared by Student’s *t*-test using Microsoft Excel. Results were considered significant when *p*-value ≤ 0.05.

**Figure 1:**
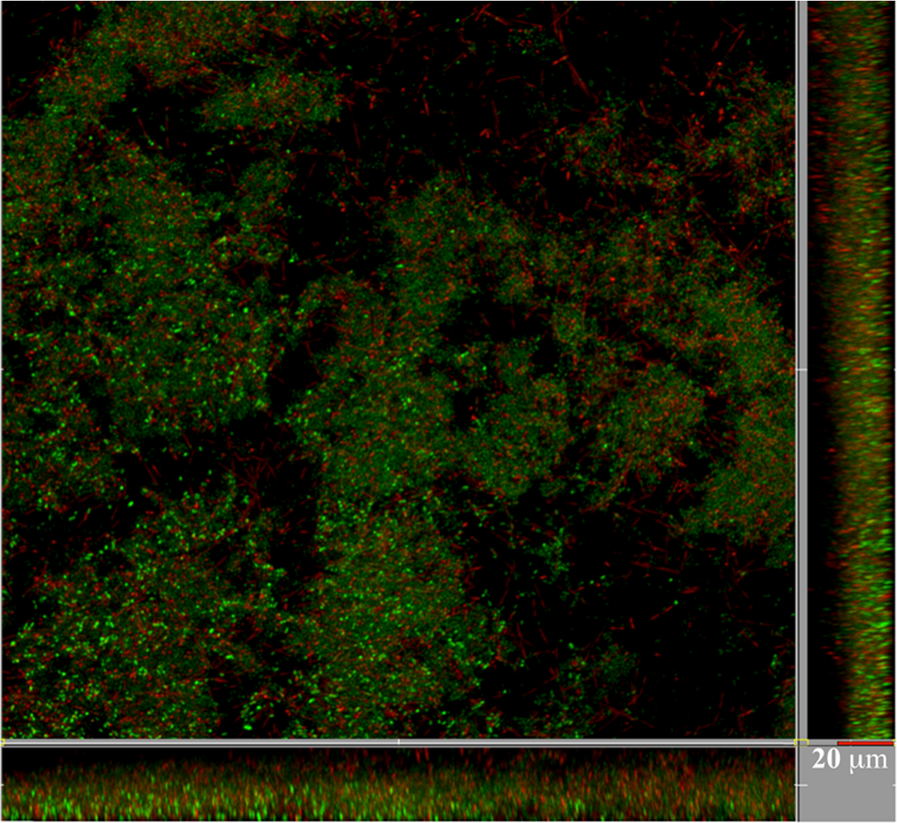
**Visualisation of multispecies oral biofilm using confocal microscopy.** Staining was performed with Live/Dead BacLight bacterial viability kit. Live and dead cells are visualised in green and red, respectively.

**Figure 2:**
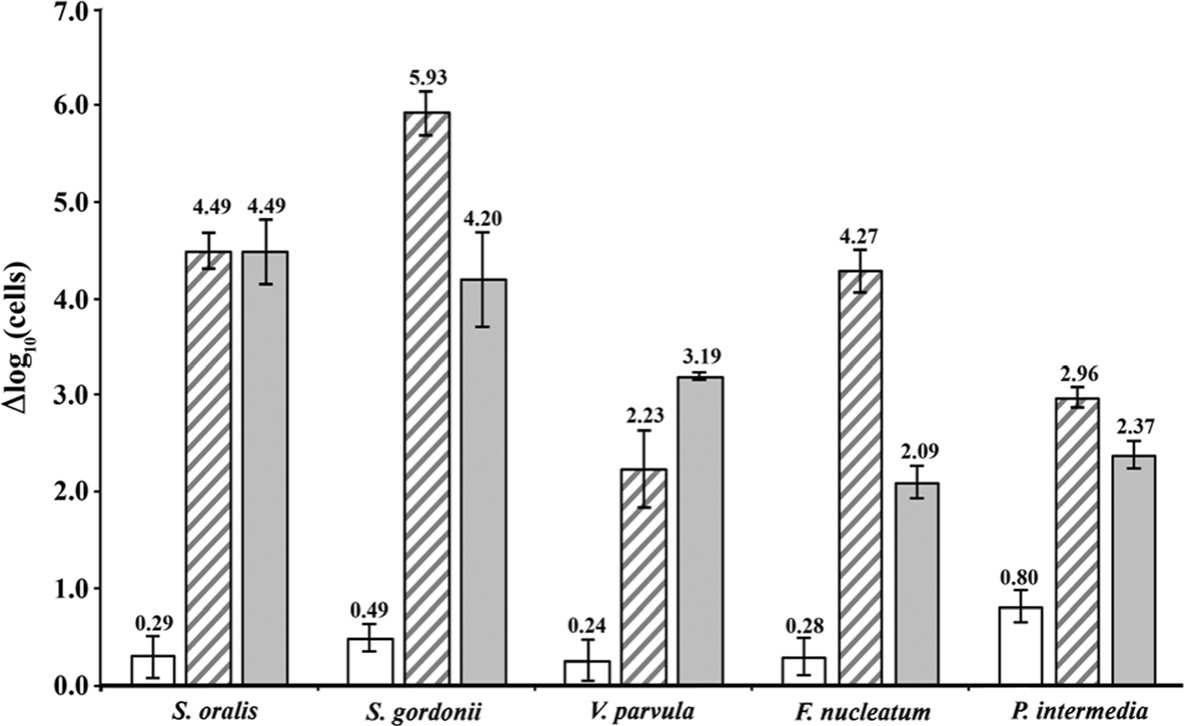
**Optimisation of PMA methodology.** Pure planktonic cultures were subjected to heat- and CPC-killing conditions and treated with 100 μM PMA. Amount of live cells (qPCR-PMA) was subtracted from the total amount of cells (qPCR) to obtain the number of dead cells (Δlog_10_ cells). Bars indicate the mean values in the negative control (empty bars), heat-killed cells (striped bars) and CPC-killed cells (grey bars).

Error bars in Figures 1 and 2 represent standard deviations calculated from at least four independent replicates.

## Results

### Biofilm formation

The confocal laser scanning microscopy (CLSM) analysis of biofilms formed by the five species showed an average thickness of 18.83 ± 5.37. In addition, we observed the characteristic structures of a biofilm, such as voids and mushroom shaped structures (Figure 1). Culture plate counts of the five species present in 4-day multispecies biofilms are shown on Table 3, confirming that all of them form part of this structure.

**Table 3:** Counts by culture and qPCR (mean and standard deviation) of total cells present in 4-day old oral multispecies biofilms

	**Culture**		**qPCR**	
**Bacterial species**	**Log CFU (mean)**	**Standard deviation**	**Log cells (mean)**	**Standard deviation**
*S. oralis*	6.37	0.18	7.40	0.24
*S. gordonii*	6.19	0.23	6.66	0.36
*V. parvula*	6.75	0.33	7.40	0.20
*F. nucleatum*	5.95	0.26	6.64	0.19
*P. intermedia*	6.83	0.20	6.92	0.19
Total mean	6.42	0.24	7.00	0.23

### qPCR standarization

For counts of each of the species present in the multispecies oral biofilm, conditions were standardized for quantitation by qPCR using pure cultures of the five species. The primers, specific probes and the size of the amplicons for each species are listed on Table 1. The melting temperature was 65°C for *S. oralis*, 58°C for *P. intermedia*, and 60°C for *S. gordonii*, *V. parvula* and *F. nucleatum*. The qPCR calibration curves are highly accurate with linearity values of 0.99 and efficiencies greater than 0.91 along a curve of 8 log units (Table 2).

### Optimization of the PMA protocol on pure culture of the five species

The conditions for discriminating between live and dead cells of the five species were established using pure planktonic cultures of each species.

A cell suspension was prepared containing between 10^7^ and 10^8^ CFU/ml, depending on the species. These cells were used as a negative control (untreated cells), positive control (heat-killed cells) and the sample treated with 0.07% CPC in water (w/w) for 1 min (CPC-killed cells).

The PMA concentrations and incubation and light exposure times were optimised by exposing the samples to different PMA molarities (50, 100 and 200 μM) for 5, 10, 15 and 30 min, followed by different light-exposure times (5, 10 and 30 min). The graph shown in Figure 2 was prepared by using the optimal conditions for the five species, 100 μM of PMA for 10 min and 5 min of light-exposure. In this figure, the Δlog_10_ cells are shown (logarithm of the dead cells), calculated by subtracting the estimation of the number of live cells, calculated by amplification using qPCR plus PMA, from the logarithm of the total number of cells, obtained by qPCR. Figure 2 shows that the number of dead bacteria before treatment with heat and CPC is low (empty bars), and we also observed that mortality values of higher than 3 or 5 orders of magnitude could be detected in *S. oralis*, *S. gordonii* and *F. nucleatum* with heat and with CPC. For the species *V. parvula* and *P. intermedia* mortality values lower to 3 log were obtained. Moreover, higher mortality when using 0.07% CPC for 1 min (grey bars) was only detected with this technique in *V. parvula.*

### Application of the PMA protocol on multispecies biofilms

The PMA protocol based on pure planktonic cultures of the 5 species was applied to multispecies oral biofilms (consisting of the 5 species). Bacterial counts was checked for each species on each disc by culture and qPCR, and the range was 8.91*10^5^ – 6.76*10^6^ in culture plate, depending on the species (Table 3).

To differentiate between live and dead cells using qPCR-PMA the biofilms were labelled as untreated biofilms (negative control) or CPC-killed biofilms. The latter, similarly to the CPC-killed cells, were exposed to 0.07% CPC in water (w/w) for 5 min to reach mortality levels similar to those obtained previously with heat-killed planktonic cells.

**Figure 3:**
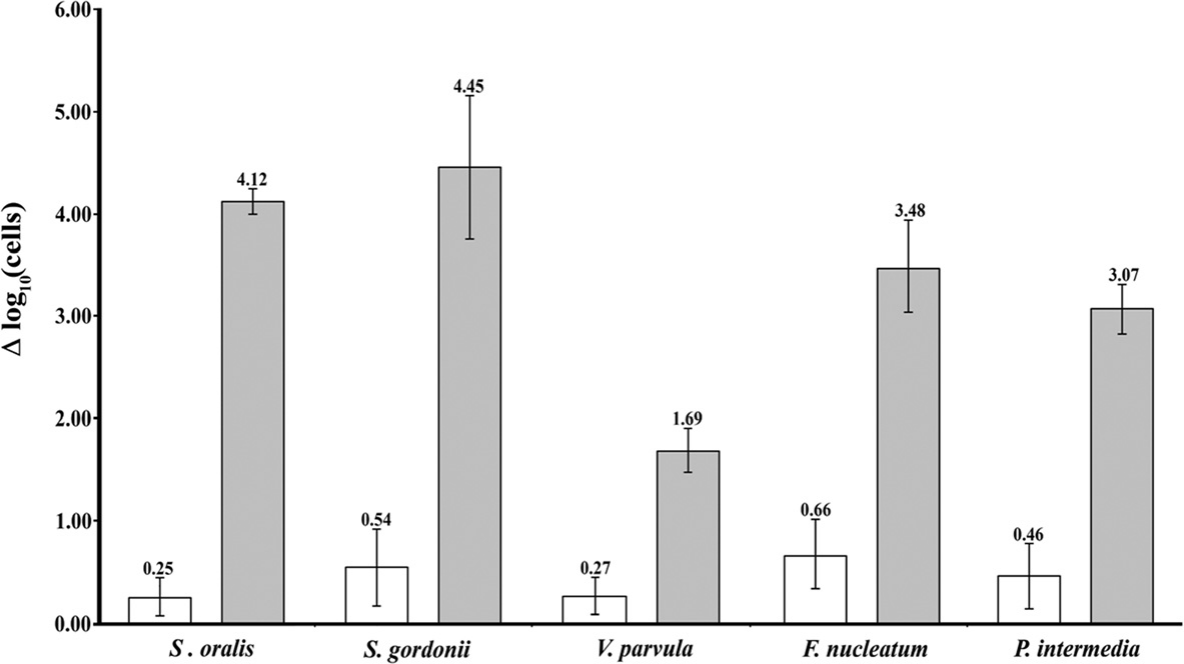
**Use of PMA on multispecies biofilms.** Oral multispecies biofilm comprising by *S. oralis, S. gordonii, V. parvula, F. nucleatum* and *P. intermedia* were exposed to CPC and treated with 100 μM PMA. The number of dead cells (Δlog_10_ cells) was found by calculating the difference between the amount of total cells (qPCR) and the amount of live cells (qPCR-PMA). Bars indicate the mean values for each species in a biofilm not exposed to CPC (empty bars) and a CPC-killed biofilm (grey bars).

Mortality (Δlog_10_ cells) was calculated similarly to that which was performed for planktonic cultures, the difference between the logarithm of total cells (qPCR) and the logarithm of live cells (qPCR-PMA) and their results are shown on Figure 3. Mortality values lower than 1 log were detected in the negative controls, where *F. nucleatum* had the greatest mortality in the biofilm after four days (white bars).

After biofilms were treated with CPC for five minutes, an increase in dead cell counts was observed: 4.12 ± 0.11 and 4.45 ± 0.61 log in *S. oralis* and *S. gordonii*, respectively. We could also detect a mortality level of *F. nucleatum* and *P. intermedia* that was higher than 3 log (3.48 ± 0.39 and 3.07 ± 0.17, respectively). Finally, in *V. parvula* only 1.69 ± 0.18 log viability reduction was obtained when using 0.07% CPC on the biofilm (Figure 3, grey bars).

## Discussion

In the present study we have optimised the conditions for qPCR to be used together with PMA (qPCR-PMA) for discriminating between the live and dead cells present in pure planktonic cultures containing five oral bacterial species. We also have proven that qPCR-PMA allows us to determine live and dead cells grown in cellular suspension and that have been previously treated with a 0.07% w/w CPC solution. Lastly, we have also shown that this methodology can be used to quantify the live and dead cells present in a multi-species oral biofilm, before and after treatment with an antiseptic molecule.

We have used PMA to discriminate between live and dead bacteria with the qPCR technique, since several authors have compared the effectiveness of this molecule with ethidium monoazide (EMA) (Nocker et al. 2006; Taskin et al. 2011; Yáñez et al. 2011; Loozen et al. 2011). In these studies PMA was determined more effective, because it more selectively penetrates the dead cells, therefore reducing the probability of obtaining false positives. Additionally, Loozen et al. 2011, showed PMA to be more efficient that EMA in oral bacteria.

During standardisation of this methodology (qPCR-PMA) different concentrations of PMA (50, 100 and 200 μM), incubation times in the dark (5, 10, 15 and 30 min) and light exposure times (5, 10 and 30 min) were tested. These conditions are similar to those that were used for other species, including oral bacteria (Pan and Breidt 2007; Yáñez et al. 2011; Taskin et al. 2011; Loozen et al. 2011). In our case, we established that the optimum conditions for the five species used in this study, both for planktonic and for biofilm, were: PMA 100 μM, incubation in the dark for 10 min and light exposure for 5 min.

Several studies have proven the usefulness of qPCR-PMA for discriminating and quantifying the live and dead cells present in different types of bacterial samples, considering its application to be of great importance in slow-growing or fastidious bacteria, where culture involves long periods of time and also where calculating or estimating cell survival is difficult (Kralik et al. 2010). Similarly, this technique has proven useful in samples of different origins: viral, fungal, spores and lyophilised bacterial samples (Fittipaldi et al. 2010; Vesper et al. 2008; Rawsthorne et al. 2009; Kramer et al. 2009).

In the oral cavity, there is a large number of slow-growing, fastidious anaerobic species. Quantification of these species using culture techniques involves long periods of time, the inconveniences of working with anaerobes and the use of specific culture media. In addition, in the mouth these bacteria mainly grow in the form of a biofilm, either supragingival or subgingival (dental plaque), which makes counts using traditional culture methods difficult, since it is pointed out in several studies that many biofilm pathogens are viable but not routinely culturable (Sun et al. 2008; Wolcott and Dowd 2008).

To our knowledge, this is the first research using the qPCR-PMA technique to analyse the live and dead cells present in a multi-species oral biofilm. These biofilms are formed *in vitro*, using five species: three primary colonisers: *S. oralis*, *S. gordonii* and *V. parvula;* one intermediate coloniser: *F. nucleatum* and one late coloniser: *P. intermedia.* Given the complexity of biofilms, the conditions for implementing this methodology were optimised using pure planktonic cultures of each species. Also, the standardisation and optimisation of this methodology was done with the idea that the five species would subsequently be treated in the same conditions when forming part of the multi-species biofilm.

All bacteria were analysed in their exponential growth phase, since in theory, all bacteria were alive. In fact, when calculating the difference between qPCR and qPCR-PMA, our results prove that, in the samples that were not heat treated, the number of dead cells is close to zero for all species (value lower than 10 cells) (Figure 2). Only *P. intermedia* yields a slightly higher value, which may be a result of handling during sample treatment, considering that this pathogen is a strict anaerobe.

When observing the results obtained with heat-killed planktonic cells, we can could see reductions between 2.23 and 5.93 log; on the other hand, the viability reductions obtained with CPC were between 2.09 and 4.49 log. These discrepancies may be caused by the specific characteristics of the membrane of each species, since CPC would act at this level to cause cell death (Mandel 1988) and heat treatment is more aggressive and would tend to more easily disorganise the membranes.

When control biofilms (not treated with CPC) were analysed, it was observed that the number of dead cells (Δlog_10_ cells) in the five species was lower than 10 (Figure 3, white bars). In these conditions, *F. nucleatum* was the one with greatest mortality (0.66 ± 0.28), similar to that which was observed when the biofilms were analysed using CLSM and Live/Dead BacLight bacterial viability kit (Figure 1).

The highest mortality detected in the CPC-killed biofilms (Figure 3, grey bars) was found in *S. gordonii* and *S. oralis* (4.45 and 4.12 log, respectively) with mortality values similar to those obtained when using planktonic cells (Figure 2, striped bars). These species were also generally the ones that better responded to treatment with PMA, when they were heat-killed (in planktonic state) and CPC-killed (planktonic and biofilm). Besides the discrepancies described for this methodology depending on the species, this difference could also be due to the fact that both have a larger amplicon, compared to the other 3 species, and that their targets are single-copy sequences (Table 1). It has been pointed out that both of these characteristics favour the efficiency of this technique, both with EMA and with PMA (Soejima et al. 2008; Contreras et al. 2011). Something similar could be argued when comparing the differences observed between *F. nucleatum* and *P. intermedia* and *V. parvula*.

As other authors have described (Chang et al. 2010; Løvdal et al. 2011; Yáñez et al. 2011), mortality detected by plate counts is generally greater than by the qPCR-PMA methodology. Our results have the same characteristics in planktonic cells as well as in biofilm, in some species with a 1.5 log difference (*S. oralis* and *S. gordonii*) and in others 3–4 log, approximately (*V. parvula*) (Figure 2). This discrepancy has been attributed to the fact that PMA can detect as live cells those that, although they have lost their metabolic activity, their membranes are still kept intact, resulting in their inability to grow on a plate. The same thing occurs when we consider viable but non-culturable bacterial cells (VBNC), which when using qPCR-PMA can be detected as live, but cannot grow on a plate (Nocker and Camper 2009). In addition, the loss of linear correlation has been described between CFU and cells estimated by qPCR-PMA when the “dead cell/live cell” ratio is over 4 (Pan and Breidt 2007). In our experiments we observe this phenomenon both for heat-killed cells and for CPC-killed cells, with mortalities of more than 4 orders.

In short, this study shows the usefulness of the qPCR-PMA methodology for discriminating between live and dead cells in a multi-species oral biofilm, with the idea that this is the form of life that bacteria adopt when they are found in their natural habitat. From this perspective, this methodology (qPCR-PMA) can solve problems related to the overestimation of bacterial counts due to the presence of extracellular DNA in biofilm matrix when using qPCR alone. Also, it makes viable cell counts more accurate, since it has been described that some pathogens that are present in a biofilm are not routinely culturable.

Furthermore, the objective of testing the effectiveness of this methodology by using CPC on a multi-species biofilm, was to determine if the qPCR-PMA technique could be used in general to determine the effectiveness of the action of mouthrinses containing this molecule or other quaternary ammoniums (chlorhexidine) on this kind of biofilm. Our results support the use of this technique, since usually the values of viability reduction in this type of trial is approximately 1–2 orders (Guggenheim and Meier 2011), and our results fall within these detection limits. Similarly, this methodology could be useful in the analysis of clinical samples, since its bacterial counts are normally within the application range of this trial (Loozen et al. 2011).

## Competing interests

The authors declare that they have no competing interests.
